# Reach and impact of you and me, together vape-free: a school-based E-cigarette prevention curriculum for elementary, middle, and high school students

**DOI:** 10.1016/j.pmedr.2026.103453

**Published:** 2026-03-18

**Authors:** Devin McCauley, Holly Lung, Michael Baiocchi, Scott Gerbert, Bonnie Halpern-Felsher

**Affiliations:** a REACH Lab, Division of Adolescent Medicine, Department of Pediatrics, Stanford University, Palo Alto, CA 94304, United States; b Department of Epidemiology and Population Health, Stanford University, Palo Alto, CA 94304, United States

**Keywords:** E-cigarettes, Tobacco prevention, Program evaluation, Youth, Education

## Abstract

**Objectives:**

This evaluation assesses the reach and impact of You and Me, Together Vape Free – an e-cigarette prevention curriculum developed by the Stanford REACH Lab with versions for elementary, middle, and high school students.

**Methods:**

Reach was measured via educator trainings, Data Dashboard registrations, and Google Analytics. Embedded in the Dashboard were brief pre- and post-curriculum surveys in which participating students reported their perceptions of e-cigarette harms, awareness of targeted marketing, refusal skills, and intentions. Data were collected between September 2022 and June 2025 in the U.S. Descriptive statistics were applied to examine pre- and post-surveys.

**Results:**

From September 2022 to April 2025, 2236 educators have been trained on You and Me, and 272,573 unique visitors accessed the curriculum webpage. The Data Dashboard indicates 230 educators and 5610 students registered for the elementary school version, and 920 educators and 60,822 students registered for the middle/high school versions. There were positive changes for all items related to perceptions of e-cigarette harm and targeted marketing by the tobacco industry.

**Conclusions:**

You and Me, Together Vape-free has been adopted throughout the United States, with positive implications for students' e-cigarette perceptions and awareness of targeted marketing. Further studies are needed to assess impact on long-term e-cigarette use outcomes.

## Highlights

• The You and Me curriculum Has been implemented in all 50 U.S. States.
• Has reached at minimum 2000 K-12 educators and 330,000 students.
• Is linked with immediate positive changes in students' e-cigarette perceptions.
• Is linked with greater awareness of tobacco industry targeted marketing.

## 1 Introduction

E-cigarettes are the most commonly used tobacco product among United States adolescents (Jamal et al., 2024), with national surveys estimating that over 1.5 million middle and high school students have used e-cigarettes in the past 30 days (Jamal et al., 2024). Adolescent e-cigarette use is associated with numerous health concerns, including risk for cardiovascular and respiratory disease (Rose et al., 2023), depression (Javed et al., 2022), nicotine addiction (Glantz et al., 2022), and use of other tobacco and cannabis products (Hair et al., 2021; McCauley et al., 2024).

Reasons for adolescent e-cigarette initiation and use include attractive youth-oriented flavors (King, 2022); targeted marketing toward young people (Padon et al., 2017); social pressures to use (Bernat et al., 2018; Jha and Kraguljac, 2021); and misperceptions about health harms and benefits, including beliefs that e-cigarettes are not addictive and relieve stress (Bernat et al., 2018; Jha and Kraguljac, 2021). The prevalence of adolescent e-cigarette use and associated health harms have prompted the development of curriculums and educational programs to reduce initiation and use (Baker et al., 2022; Liu et al., 2026). Many programs are designed for school implementation since school personnel are uniquely positioned to reach large groups of adolescents (Liu et al., 2026). These school-based educational programs typically include lessons designed to increase adolescents' knowledge of e-cigarette-related health harms, raise awareness of targeted marketing, and equip adolescents with coping strategies and refusal skills (Baker et al., 2022; Liu et al., 2026). Participation in school-based e-cigarette prevention programs is associated with improved e-cigarette knowledge, perceptions, intentions, and refusal skills (Baker et al., 2022; McCauley et al., 2023; Gaiha et al., 2021a).

The You and Me, Together Vape-Free curriculum (abbreviated You and Me; https://med.stanford.edu/tobaccopreventiontoolkit/you-and-me-together-vape-free-curriculum.html), part of the Stanford REACH Lab's larger Tobacco Prevention Toolkit (Gaiha et al., 2021a; Gaiha et al., 2021b) (https://med.stanford.edu/tobaccopreventiontoolkit.html), launched in 2022. You and Me has several unique features compared to other curriculums (Liu et al., 2026), including being free and available online; offering free educator trainings; and being developed using a community-based participatory research approach (Faggiano et al., 2014; Israel et al., 1998) with input from physicians and addiction specialists, adolescents, parents, and educators to ensure the curriculum remains based in current evidence and relevant to today's youth. You and Me is theory-based, informed by the Social Influence Model (Bandura, 1977), Theory of Planned Behavior, and the Positive Youth Development framework (Shek et al., 2019) to promote positive behavior change and empower adolescents to make healthy decisions around e-cigarette use in social contexts (Liu et al., 2026; Gaiha et al., 2021b). Additionally, You and Me has clearly delineated content that includes updated information, and a unique data dashboard that allows educators, in real time, to evaluate the impact of the instruction and re-teach concepts if necessary.

The goals of You and Me are to increase adolescents' knowledge about e-cigarettes and the harms they can cause; help adolescents gain awareness of tobacco industry targeted marketing strategies; help adolescents gain skills to refuse experimentation and use of e-cigarettes; and ultimately prevent and reduce e-cigarette use of any type, including nicotine and cannabis/THC. You and Me includes separate versions developed for elementary, middle, and high school students. The middle and high school curriculums include six lessons: (1) “Full of Potential: Your Brain Nicotine-Free”; (2) “Healthy Body, Healthy You-th: Effects of E-cigarettes on The Body”; (3) “What a Waste! Impact of Cigarettes and E-cigarettes on the Environment”; (4) “Don't Be Played! How Tobacco Marketing Targets You-th”; (5) “Be Your Strength: Stress, Coping, and Wellness”; (6) “Can't Be Missed: Cannabis & You-th.” The elementary school curriculum has two lessons: (1) “Take Care of Your Body” (e-cigarette overview and health effects) and (2) “Don't Be Fooled” (marketing, the environment, stress and coping, and refusal skills). Lessons are designed to be implemented by school educators or other personnel (e.g., nurses, counselors) and include (a) didactic lessons presented using Canva slides; (b) classroom discussions and activities; (c) take-home discussion guides for students and their parents/guardians; and (d) resources for students and parents/guardians. Voluntary and free educator trainings to use You and Me are provided by the Stanford REACH Lab team. These 90–120 min online (via Zoom) educator trainings have been well received by educators (Lazaro et al., 2021), and demonstrated to improve educators' knowledge about e-cigarettes and confidence in implementing You and Me (Liu et al., 2025).

Previous studies have evaluated parts of the Tobacco Prevention Toolkit (Gaiha et al., 2021a) as well as a pilot version of You and Me (McCauley et al., 2023), finding positive changes in adolescents' knowledge, perception of harm, refusal skills, and intentions to use. Prior evaluations informed the topics, learning objectives, lesson content, activities, and visual design of the current version of You and Me. However, the extent of You and Me's use and its impact have yet to be evaluated. As such, this evaluation examines the general reach of the You and Me, Together Vape Free curriculum within the United States, including the extent to which the e-cigarette prevention curriculum is being used by schools, educators, and students. Additionally, we evaluate whether You and Me changes students' e-cigarette perceptions of harm, perceptions of targeted marketing, knowledge, refusal skills, and intentions to use.

## 2 Methods

### 2.1 Study design and population

You and Me, Together Vape-Free is freely accessible to anyone. Embedded in the Curriculum is a Data Dashboard through which educators have the option to register that their school is using the curriculum, thereby providing information on the number and types of schools (e.g., elementary, middle, high schools) as well as number of classrooms and educators within each school using You and Me. If schools choose to, they then give their students the QR code to complete a five-minute pre- and post-curriculum survey that is embedded within the curriculum. These are the data that are within this evaluation for this particular convenience sample. As such, we did not specifically recruit educators or students for this evaluation, but are analyzing the data from the Dashboard. To support accessibility of curriculum materials, dashboard registrations and educator trainings are not mandatory but encouraged. Stanford University's IRB deemed the study as program evaluation and not human subjects research, and thus was not subject to further IRB review.

### 2.2 Measures

#### 2.2.1 Curriculum reach measures

The reach of You and Me was estimated through the following sources: (1) Data Dashboard registrations; (2) number of teachers trained on You and Me through formal trainings and presentations; and (3) Google Analytics. Note that estimates derived through each approach are not intended to be summative but rather represent distinct reach metrics.

Although You and Me launched in 2022, the Data Dashboard did not go live until October 2023. Information collected from registered educators on the Data Dashboard include which curriculum version was implemented (i.e., elementary or middle/high school), grade levels taught, school, and state. Number of students completing pre- and post-curriculum surveys are also logged within the Data Dashboard. See Table 1.

**Table 1:** You and Me, Together Vape-Free Data Dashboard embedded survey completions by students and registrations by educators between October 2023 and June 2025.

Student Data	Elementary School Version		Middle/High School Version	
Grade Level	Pre n (%)	Post n (%)	Pre n (%)	Post n (%)
K	16 (0.3)	9 (0.2)	8 (0.0)	2 (0.0)
1	22 (0.4)	13 (0.3)	18 (0.0)	1 (0.0)
2	19 (0.3)	12 (0.3)	34 (0.1)	3 (0.0)
3	164 (2.9)	113 (2.5)	43 (0.1)	17 (0.0)
4	499 (8.9)	244 (5.3)	85 (0.1)	5 (0.0)
5	1290 (23)	731 (15.9)	204 (0.3)	114 (0.3)
6	1433 (25.5)	864 (18.8)	14,694 (24.2)	8669 (24.2)
7	2167 (38.6)	2613 (56.8)	18,007 (29.6)	10,027 (28)
8	–	–	10,219 (16.8)	6600 (18.5)
9	–	–	11,349 (18.7)	6891 (19.3)
10	–	–	3324 (5.5)	1795 (5)
11	–	–	1431 (2.4)	817 (2.3)
12	–	–	1377 (2.3)	821 (2.3)
College +	–	–	29 (0.0)	5 (0.0)
**Total**	**5610**	**4599**	**60,822**	**35,767**
**Educator Registrations**	Elementary School Version		Middle/High School Version	
Educators	230		920	
Schools	282		1394	
U.S States	37		50	
Countries/Territories Outside of the U.S.	2		3	

Note. Countries/Territories outside the United States include Guam, Cayman Islands, and Argentina. Some educators (e.g., counselors, district level staff) implemented the curriculum at multiple different schools.

The Stanford REACH Lab documented the number of attendees who completed trainings for You and Me between September 2022 and April 2025. To estimate the number of students reached, we multiplied each trained educator by 150. This estimation method is informed by surveys with educators indicating that 40% anticipate they will educate at least 150 students using You & Me curriculum materials within the next year, and 20% expecting to reach over 300 students. (Liu et al., 2025)

Google analytics for the You and Me website include the total unique users of the You and Me website between September 2022 and July 2025, including the number of users who visited the website through directly entering the URL, organic searches, referrals from other websites or email links, and social media links. We also examined total page views for You & Me website materials overall and by curriculum type.

#### 2.2.2 Student survey measures

Separate surveys were designed for (a) elementary school students and (b) middle and high school students combined. Students provided no identifying information, sociodemographic characteristics, or substance use behaviors.

Students completed items related to (a) perceptions of e-cigarette health harms and addictiveness, (b) perceptions of tobacco industry targeted marketing, (c) healthy coping strategies, (d) refusal skills, and (e) vaping goals. The items used were based off of previous evaluations of the Tobacco Prevention Toolkit and evaluations of other similar curriculums (Baker et al., 2022; McCauley et al., 2023; Gaiha et al., 2021a). See Table 2, Table 3 for complete item and scale information for elementary and middle/high school versions of the surveys, respectively.

**Table 2:** You and Me, Together Vape-Free elementary version pre- and post-survey assessment results.

Perceptions of Health Harms^a^	Pre M (SD)	Post M (SD)	Cohen's d
**Imagine you vape nicotine once in a while…**			
*How would this affect your body?*	3.23 (1.1)	3.45 (1.1)	0.19
*How likely are you to become addicted?*	3.45 (1.2)	3.62 (1.1)	0.15
**Imagine you vape nicotine daily…**			
*How would this affect your body?*	4.61 (0.82)	4.72 (0.69)	0.15
**Healthy Coping Strategies**^b^	Pre M (SD)	Post M (SD)	Cohen's d
*I know healthy ways to cope with stress*	3.40 (1.3)	3.82 (1.2)	0.33
**Perceptions of Marketing**	Post n (%)	Post n (%)	Cohen's h
***Why do you think that tobacco companies try to trick kids to start smoking or vaping?***			
*They want to make more money*	3758 (85.4)	3490 (90.6)	0.16
*They don't trick kids*	219 (4.9)	73 (1.9)	0.17
*They like kids*	154 (3.5)	132 (3.4)	0.01
*They think kids should try tobacco*	269 (6.1)	155 (4.0)	0.09
**Knowledge**	Post n (%)	Post n (%)	Cohen's h
***Can vaping hurt other people too?***			
*Yes, vaping can hurt other people*	3499 (79.6)	3475 (90.7)	0.32
*No, vaping cannot hurt other people*	252 (5.7)	109 (2.8)	0.19
*I don't know*	647 (14.7)	246 (6.4)	0.28
**Are nicotine vapes safer than cigarettes?**			
*No, vapes are not safer than cigarettes*	2038 (46.4)	2697 (70.2)	0.49
*Yes, vapes are safer than cigarettes*	772 (17.6)	387 (10.1)	0.22
*I don't know*	1585 (36.1)	757 (19.7)	0.37
**Vaping Goals**	Post n (%)	Post n (%)	Cohen's h
**I want to…**			
*Never use*	3845 (87.0)	3464 (90.0)	0.09
*Cut back my vaping*	73 (1.7)	46 (1.2)	0.04
*I want to quit completely*	125 (2.8)	92 (2.4)	0.03
*I want to change what I vape*	42 (0.9)	43 (1.1)	0.02
*I have not decided yet*	333 (7.5)	204 (5.3)	0.09

Note. Results based on 4471 pre-surveys and 3894 post-surveys collected between October 2023 and January 2025.

a Scale was 1 to 5 blocks.

b 1 (I don't know any other ways to cope with stress) to 5 (I know lots of ways to cope with stress). For Perceptions of Marketing and Knowledge questions on the elementary school surveys, the first response provided in the table was scored as the correct answer, with all others being scored as incorrect. Regarding the question “Are nicotine vapes safer than cigarettes?”, please see comment in the limitations section.

**Table 3:** You and Me, Together Vape-Free middle school and high school version pre- and post-survey assessment results.

Perceptions of Health Harms	Pre M (SD)	Post M (SD)	Cohen's d
**Imagine you vape nicotine daily…**			
*How harmful would this be for your health?*^a^	4.46 (0.79)	4.56 (0.76)	0.13
*How likely are you to become addicted?*^b^	4.22 (1.18)	4.35 (1.09)	0.11
**Imagine you vape nicotine occasionally…**			
*How harmful would this be for your health?*^a^	3.59 (1.00)	3.77 (0.97)	0.18
*How likely are you to become addicted?*^b^	3.38 (1.13)	3.55 (1.09)	0.15
*How harmful are e-cigarettes to the environment?*^a^	3.90 (0.96)	4.17 (0.90)	0.29
*Nicotine vapes are safer than cigarettes*^c^	2.47 (1.04)	2.22 (1.07)	0.24
**Perceptions of Marketing**	Pre M (SD)	Post M (SD)	Cohen's d
**Tobacco and Vaping (E-cigarette) Companies Target…**^c^			
*Youth*	3.75 (1.09)	4.09 (1.03)	0.31
*Adults*	3.41 (0.97)	3.43 (1.00)	0.02
*Black and Brown communities*	3.02 (1.00)	3.43 (1.05)	0.39
*LGBTQ+ communities*	2.86 (0.98)	3.18 (1.04)	0.32
**Refusal Skills**	Pre M (SD)	Post M (SD)	Cohen's d
**How hard would it be for you to refuse, or say “no” to a friend who offered you a…**^d^			
*E-cigarette/vape?*	1.61 (0.99)	1.62 (0.97)	0.00
*Cigarette to smoke?*	1.57 (0.96)	1.59 (0.95)	0.02
**Vaping Goals**	Pre n (%)	Post n (%)	Cohen's h
**I want to…**			
*Never use*	34,160 (88.1)	16,428 (86.7)	0.04
*Cut back my vaping*	745 (1.9)	369 (1.9)	0.00
*I want to quit completely*	1158 (3.0)	774 (4.0)	0.06
*I want to change what I vape*	351 (0.9)	319 (1.7)	0.07
*I have not decided yet*	2371 (6.1)	1053 (5.6)	0.02

Note. Results based on 39,189 pre-surveys and 19,174 post-surveys collected between October 2023 and January 2025.

a Scale was 1 (Not at all harmful) to 5 (Extremely harmful).

b 1(Not at all likely to become addicted) to 5 (Extremely likely to become addicted).

c 1 (Strongly disagree) to 5 (Strongly agree).

d 1(Very easy) to 5 (Very hard).

For the elementary school version of You and Me, survey responses for perception items were represented by an increasing number of graphic blocks, from one to five, with instructions indicating a higher number of blocks represented greater levels of perceived harm. For the remaining items in the survey, visual representations such as emojis, including smiley and sad faces, as well as thumbs up/thumbs down, were integrated alongside the text response options. The use of graphics and emojis has been implemented to support understanding and engagement of survey measures among elementary age participants (Massey, 2022).

### 2.3 Statistical analysis

Pre-post survey responses were analyzed by curriculum type: (1) elementary school and (2) middle school and high school combined. Middle and high school curriculums are analyzed together due to the similarity in curriculum content and pre-post survey measures. Pre-post surveys did not include any identifying information or codes for linking student responses; therefore, descriptive statistics (i.e., means, standard deviations, proportions, and effect sizes) were applied to assess differences in pre and post survey responses. Unpaired *t*-tests were not applied because pre- and post-survey samples were non-independent and resulting standard errors would have been artificially smaller than if the data allowed for repeated measures analysis.

In a set of exploratory analyses, we applied linear fixed-effect models to account for variation in pre-post survey responses by student grade level (Muff et al., 2016). This approach was applied only to middle/high school survey results due to limited distribution of elementary school responses by grade level. Students were categorized into the following four school grade level groups: 1 = 6th grade; 2 = 7th and 8th grade; 3 = 9th and 10th grade; 4 = 11th grade and 12th grade. Grade level groupings were chosen to allow for differentiation within middle and high school grades. The outcome was average change in each study variable from pre to post survey. School grade level was included as a fixed effect to account for variation in pre-test scores by grade level, and also a moderator to assess differences in pre-post score changes by grade level (Brown, 2021). An example equation is below:

$$\;\text{TargetedMarketing}\_{\text{Youth}}_{\mathrm{it}}=\beta_{0}+\beta_{1}{\text{Treatment}}_{\mathrm{it}}+\beta_{2}\text{Grade}\_{\text{Level}}_{\mathrm{it}}+\beta_{3}{\text{Treatment}}_{\mathrm{it}}*\text{Grad}e\_{\text{Level}}_{\mathrm{it}}+\epsilon_{\mathrm{it}}$$

where *Targeted Marketing_Youth* is the average outcome score for a student *i* at time point *t*; $\beta_{0}$ is the intercept function representing average pre-test outcome score for 6th grade students; $\beta_{1}$ is the coefficient function for Treatment, representing the estimated association between Treatment (0 = pre-survey, 1 = post-survey) and the outcome score; $\beta_{2}$ is the coefficient function for Grade_Level, representing the estimated association between Grade level group and the outcome score (where grade is modeled as a categorical variable); $\beta_{3}$ is the coefficient function for the interaction between Treatment and Grade_Level, reflecting the difference in the association between treatment and the outcome variable for each grade level group related to students in 6th grade. The error term, $\epsilon_{\mathrm{it}}$, represents the residual error for individual *i* at time *t.* In this exploratory analysis, the parameter of interest is $\beta_{3}$ since it is suggestive of differences in reaction to the treatment by grade level category. An important limitation of the data collection for this study causes issues with this model (e.g., artificially lowers the standard error estimates) and is captured by the index *i*; because individual-level identifiers were not collected, we treat the pre- and post- samples as independent and thus a particular student has two different indexes if they appear in both the pre-survey period and the post-survey period.

Intra-class correlations (ICC's) for all models were low (<0.10), indicating low clustering of data by school grade level (Bolger and Laurenceau, 2013). Therefore, no random effects were included. Models were run at the student level. However, these exploratory results are included to supplement primary findings and inform future work examining heterogenous treatment effects of the You and Me curriculum by student grade level.

To examine differential rates of post-survey completion (“differential drop out”), we examined pre and post-test completion rates by grade level and geographic region, and applied a logistic regression model in which survey type (0 = pre, 1 = post) was regressed on (a) student grade (i.e., 5th through 12th grades) and (b) geographic region (i.e., 1 = Pacific West; 2 = Mountain West; 3 = Northeast; 4 = Midwest; 5 = South; 6 = Outside the United States). Data cleaning and scoring were conducted in IBM SPSS for Mac, Version 28.0 and data analyses were performed in R Studio version 4.1.2.

## 3 Results

### 3.1 Reach metrics for the you and me, together vape free curriculum

Reach metrics for Data Dashboard registrations are demonstrated in Table 1. 230 different educators from 282 different schools across 37 U.S. states registered for the elementary school version of You and Me as of July 2025, and 5610 students completed embedded pre-surveys. For the middle and high school versions, 920 different educators from 1394 schools and 50 U.S. states registered, and 60,822 students completed embedded pre-surveys. For more information regarding educator registrations by U.S. state and region, see Supplemental Table 1.

Educator training records indicate that a total of 2236 educators have completed training sessions for You and Me, Together Vape-Free between September 2022 and July 2025. Multiplying this figure by 150 yields an estimate of 335,400 students reached through these trainings.

Google analytics indicate that between September 2022 and July 2025 there were 272,573 total unique visitors for the You and Me, Together Vape-Free website. Visitors first navigated to the website via direct access (47.88%; *n* = 130,514), internet search (43.30%; *n* = 117,917), referral from another website or email link (7.23%; *n* = 19,708), and social media (1.53%, *n* = 4157). There were 418,159 total pageviews for web pages related to You and Me, including for the elementary school (*n* = 29,751), middle (*n* = 119,226), and high school (*n* = 104,176) versions.

### 3.2 Pre- and post- surveys for elementary, middle, and high school curriculums

Analytic sample sizes (ELM = 4471 pre-surveys and 3894 post-surveys; MS/HS = 39,189 pre-surveys and 19,174 post-surveys) for pre-post survey analyses are based only on Data Dashboard data provided from October 2023 until January 2025 due to survey changes that occurred after January 2025. See Supplemental Table 2 for a complete breakdown of student pre- and post-surveys by grade level for the analytic sample.

Pre- and post-survey results for students participating in the elementary school curriculum are shown in Table 2. After participating in You and Me, students demonstrated more positive responses on all three perception of harm items (*Cohen's ds* ≥ 0.15), the healthy coping item (*d* = 0.33), knowledge items related to second-hand effects of vaping (*Cohen's h* > 0.32), tobacco marketing (*h* = 0.16), and safety of e-cigarettes compared to cigarettes (*Cohen's h* = 0.49). Following You and Me, a higher proportion of students indicated they never wanted to use vapes (*Cohen's h* = 0.09), and a lower proportion responded they had not yet decided their vaping goals (*Cohen's h* = 0.09).

Pre- and post-survey scores for students participating in middle and high school curriculums are show in Table 3. Following You and Me, student responses were more positive for all six perception of harm items (*Cohen's ds* > 0.11), and three of four targeting marketing items (*Cohen's ds* ≥ 0.31). There were not meaningful differences in cigarette and e-cigarette refusal skills (*Cohen's ds* ≤ 0.02).

For students' vaping goals, a slightly higher percentage of students reported wanting to quit vaping completely following You and Me (*Cohen's h* = 0.06) or changing what they vape (*Cohen's h* = 0.07).

Results from linear mixed-effect models indicating differences in pre- and post-curriculum survey scores by student school grade level are shown in Supplemental Table 3. Supplemental Fig. 1 demonstrates differences in pre- and post- survey scores by student school grade level for (1a) perceptions of e-cigarette harm; (1b) perceptions of e-cigarette addiction risk; and (1c) perceptions of targeted marketing toward youth.

### 3.3 Post-survey completion rates

For the middle/high school version of You and Me, the post-curriculum survey completion rate was 48.9%, with highest rates of completion for students in 12th grade (59.5%) and 9th grade (58.6%) and Pacific (50.8%) and Midwest regions (53.7%). Odds of post-test completion were lowest among students in 7th and 8th grades and from the South and Northeast regions, and areas outside of the United States. See Supplemental Table 4 for complete logistic regression results.

## 4 Discussion

This study examines the reach and impact of the You and Me, Together Vape-Free curriculum, an e-cigarette prevention curriculum for elementary, middle, and high school settings. Study findings highlight that over 2000 educators have completed trainings for You and Me, and we have reached well over 300,000 students thus far through these trainings alone. Additionally, educators from all 50 U.S. states have registered for You and Me on The Data Dashboard. Because educator trainings, data dashboard registrations, and student pre- and post-curriculum surveys are not mandatory for implementing You & Me, these numbers are likely underestimates of the total educator and student reach. Results from the Data Dashboard highlight that the middle and high school versions of You and Me, Together Vape-Free were the most implemented, and most participating students were in 6th through 9th grades. This pattern is expected given these grades overlap with sensitive developmental periods for tobacco initiation (Chen et al., 2017), and are a common time for students to take health education classes during which implementation of school-based tobacco prevention curriculums typically occurs (Baker et al., 2022; Liu et al., 2026). The elementary school version was used in over 35 U.S. states with over 5000 students. Given some youth are initiating e-cigarettes as early as age seven (Chen et al., 2017), targeted vaping-prevention curriculums for elementary aged students are an important piece of broader prevention efforts.

You and Me, Together Vape-Free, across all grades/age groups, was associated with positive changes in students' perceptions of e-cigarette health harms and addiction, a finding consistent with earlier studies (e.g., McCauley et al., 2023; Gaiha et al., 2021a). Notably, examination of grade level differences in curriculum effects indicated that while younger students (i.e., 6th grade) typically perceived vaping to be more harmful to one's health relative to students in older grade levels, they perceived relatively lower risk of addiction. Grade level differences in perceptions of addiction diminished following You and Me, and highlight education around nicotine addiction as particularly important for younger students who may not fully grasp this concept (Roditis et al., 2016).

Also promising was that following You and Me, students reported higher awareness that tobacco companies target specific demographic groups with their marketing and advertising, including young people, Black and Brown communities, and LGBTQ+ communities. Given the targeted marketing of these demographic groups (Cruz et al., 2019; Richardson et al., 2015), and prevalent marketing of tobacco products in social media platforms often used by young people (Vogel et al., 2021), equipping students with knowledge and counter-marketing strategies represents an increasingly important component of e-cigarette prevention (Liu et al., 2026). You and Me appeared to have a particularly strong impact on younger students' (i.e., 6th grade) awareness that the tobacco industry targets young people, as these students demonstrated the lowest perceptions of targeted marketing of youth prior to the curriculum.

Students also perceived e-cigarettes to be more harmful to the environment following You and Me. Prior studies demonstrate that tobacco companies commonly engage in “greenwashing” in their marketing efforts to project an environmentally friendly image despite the vast environmental cost of the tobacco industry, from deforestation, to water supply, to waste disposal (World Health Organization, n.d.). Given many adolescents express concern for the environment (Drumm and Vandermause, 2023), improving students' understanding of the environmental impacts of e-cigarette manufacturing and waste may be a powerful avenue for positively supporting perceptions of e-cigarette harms and counter-marketing skills.

This evaluation has several limitations. First, data were collected from teachers and students who opted to register for the Data Dashboard; therefore, results regarding curriculum impact may not be generalizable to all curriculum users. Second, the pre- and post- surveys did not contain unique identification information, so the reported analyses treat these samples as two independent samples; thus these analyses likely understate the uncertainty in the reported estimates and therefore these results should not be used for hypothesis testing. Some regions of the U.S. completed post-tests at significantly lower rates than others, meaning pre-post analyses are also less generalizable to students from these geographic regions. Furthermore, it is unclear why some elementary school educators opted to use the middle or high school versions of You and Me when a targeted elementary school version was available. Interviewing educators could identify barriers to finding and using the developmentally appropriate version of You and Me. The survey question “nicotine vapes are safer than cigarettes” is vague and should be revised in future studies to more specifically assess nicotine and other ingredients contained in e-cigarettes versus cigarettes, as the curriculum does not explicitly compare cigarette versus e-cigarette health harms. The current study did not include a control group; therefore, the analyses are not intended to examine causality. Ongoing randomized control trials of You and Me with longitudinal data collection offer a promising opportunity for evaluating curriculum impact in a controlled setting, while also assessing heterogeneous treatment effects.

## 5 Conclusion

You and Me, Together Vape Free is estimated to have reached at least 300,000 students from the trainings alone across 50 U.S. states from September 2022 through April 2025 and is associated with changes in students' perceptions of e-cigarette harms, awareness of targeted marketing by the tobacco industry, and healthy coping skills that are aligned with an anti-e-cigarette profile. Future studies are needed to evaluate the long-term causal impact of You and Me on adolescents' patterns of use, as well as heterogeneous effects by age, sex, race/ethnicity, and geographic region.

## CRediT authorship contribution statement

**Devin McCauley:** Writing – review & editing, Writing – original draft, Methodology, Investigation, Formal analysis, Conceptualization. **Holly Lung:** Writing – review & editing, Project administration, Methodology, Investigation, Formal analysis, Data curation. **Michael Baiocchi:** Writing – review & editing, Supervision, Methodology, Formal analysis. **Scott Gerbert:** Writing – review & editing, Software, Project administration, Methodology, Investigation, Data curation. **Bonnie Halpern-Felsher:** Writing – review & editing, Supervision, Resources, Funding acquisition, Conceptualization.

## Author statement

BHF and SG conceptualized the study aims. SG, HL, and BHF oversaw design and implementation of data collection methodologies. DMM, MB, and HL devised the analytic approach. HL performed data cleaning and curation. DMM, HL, and MB conducted data analysis. DMM drafted the manuscript, and all authors revised the manuscript critically for important intellectual content. BHF funded the study. All authors approved the final manuscript for submission.

## Funding sources

This work was supported in part from a grant from California's Tobacco-Related Disease Research Program (TRDRP) of the University of California (Grant Number 271R-0043) to Bonnie Halpern-Felsher. Devin Malloy McCauley was also supported by the Stanford Maternal and Child Health Research Institute (MCHRI) and the California Tobacco-Related Disease Research Program (TRDRP) of the University of California (Grant Number T34FT8046).

## Declaration of competing interest

The authors declare the following financial interests/personal relationships which may be considered as potential competing interests: Dr. Halpern-Felsher is the Founder and Executive Director of the Stanford Tobacco Prevention Toolkit which includes the You and Me, Together Vape-free curriculum. She is also a paid expert scientist in some litigation against the e-cigarette industry and an unpaid scientific advisor and expert regarding some tobacco-related policies. The other authors have no other conflicts of interest to declare.

## Data Availability

The data that has been used is confidential.

## Appendix A Supplementary data

Supplementary material 1 Image 1

Supplementary material 2 Image 2
