# Supplementary material for: Reach and impact of you and me, together vape-free: a school-based E-cigarette prevention curriculum for elementary, middle, and high school students
Source: Prev Med Rep. 2026 Mar 18;65:103453. doi: 10.1016/j.pmedr.2026.103453 (PMC13022631; doi:10.1016/j.pmedr.2026.103453)
Supplement: Supplementary file 1 — Supplementary material 1 [file mmc1.docx]

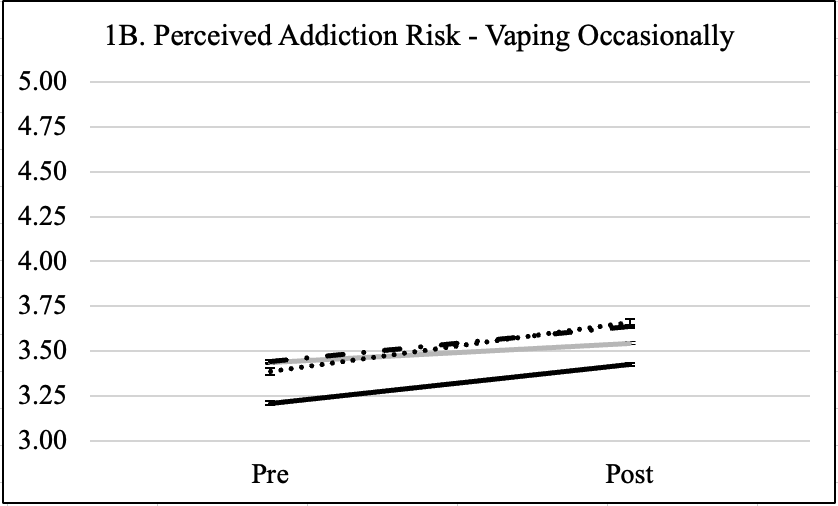
**
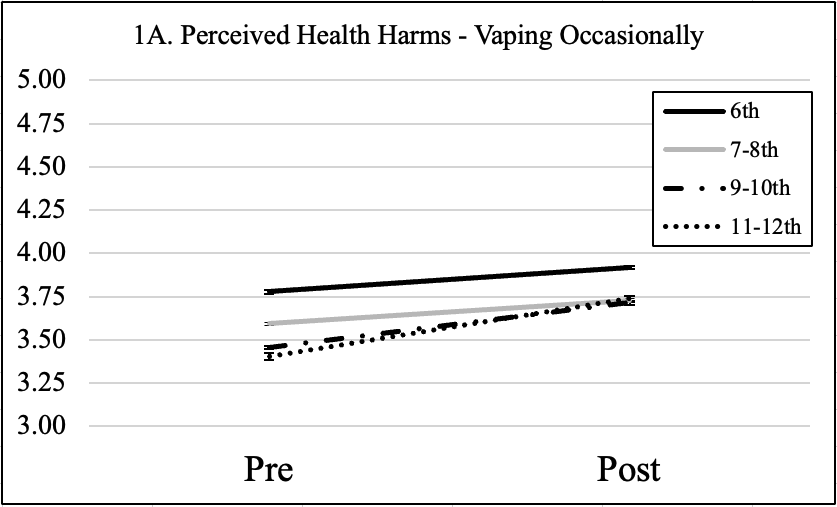
**

**Grade level**


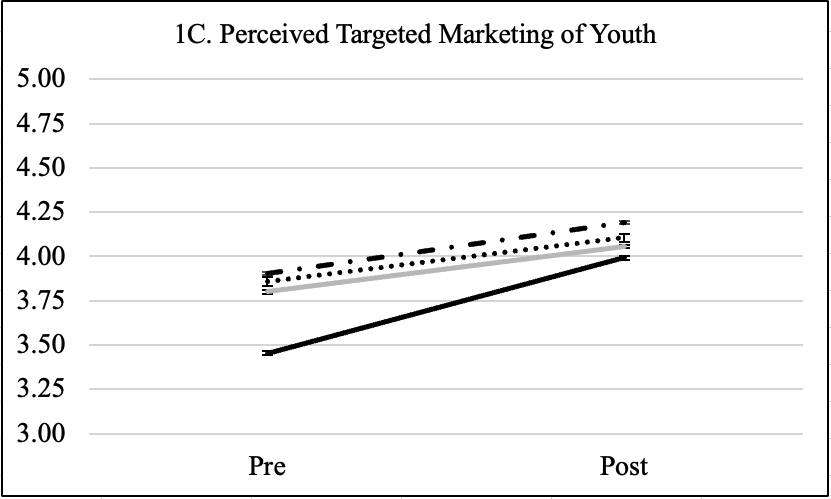
**Supplemental Figure 1.** Pre-post curriculum survey score changes as moderated by grade level for outcomes: (1A) perceived health harms from occasional vaping; (1B) perceived addiction risk from occasional vaping; (1C) perceived targeted marketing of youth. All outcomes are on 1-5 scale. Grade levels include (1) 6^th^ grade; (2) 7-8^th^ grade; (3) 9-10^th^ grade; (4) 11-12^th^ grade.
