# Supplementary material for: Reach and impact of you and me, together vape-free: a school-based E-cigarette prevention curriculum for elementary, middle, and high school students
Source: Prev Med Rep. 2026 Mar 18;65:103453. doi: 10.1016/j.pmedr.2026.103453 (PMC13022631; doi:10.1016/j.pmedr.2026.103453)
Supplement: Supplementary file 2 — Supplementary material 2 [file mmc2.docx]

SUPPLEMENTAL TABLES

**Table S1.** You and Me Data Dashboard educator registrations by geographical region, state, and curriculum version

| Region | ELM (n) | MS/HS (n) |  |  |  |
| --- | --- | --- | --- | --- | --- |
| Pacific - West | 3899 | 49640 |  |  |  |
| Mountain - West | 316 | 1668 |  |  |  |
| Northeast | 680 | 1091 |  |  |  |
| Midwest | 395 | 4827 |  |  |  |
| South | 235 | 2742 |  |  |  |
| Outside of the U.S. | 85 | 854 |  |  |  |
| **Total** | **5610** | **60822** |  |  |  |
| State/Territory | ELM  (n) | MS/HS  (n) | State/Territory | ELM  (n) | MS/HS  (n) |
| Alabama | 4 | 6 | Nebraska | 1 | 1 |
| Alaska | 1 | 3 | Nevada | 135 | 16 |
| Arizona | 2 | 86 | New Hampshire | 0 | 28 |
| Arkansas | 49 | 63 | New Jersey | 24 | 2 |
| California | 3524 | 48773 | New Mexico | 41 | 404 |
| Colorado | 128 | 919 | New York | 150 | 527 |
| Connecticut | 0 | 149 | North Carolina | 1 | 264 |
| Delaware | 0 | 541 | North Dakota | 153 | 1 |
| Florida | 3 | 125 | Ohio | 78 | 2645 |
| Georgia | 0 | 13 | Oklahoma | 0 | 3 |
| Hawaii | 118 | 52 | Oregon | 60 | 250 |
| Idaho | 10 | 62 | Pennsylvania | 37 | 84 |
| Illinois | 0 | 328 | Rhode Island | 30 | 12 |
| Indiana | 69 | 183 | South Carolina | 0 | 32 |
| Iowa | 35 | 121 | South Dakota | 25 | 77 |
| Kansas | 2 | 150 | Tennessee | 1 | 498 |
| Kentucky | 5 | 273 | Texas | 131 | 346 |
| Louisiana | 6 | 3 | Utah | 0 | 152 |
| Maine | 45 | 22 | Vermont | 392 | 52 |
| Maryland | 32 | 182 | Virginia | 3 | 292 |
| Massachusetts | 2 | 215 | Washington | 196 | 562 |
| Michigan | 21 | 1032 | West Virginia | 0 | 87 |
| Minnesota | 9 | 170 | Wisconsin | 2 | 88 |
| Mississippi | 0 | 14 | Wyoming | 0 | 1 |
| Missouri | 0 | 31 | Outside of the U.S. | 85 | 854 |
| Montana | 0 | 28 | **Total** | **5610** | **60822** |

Note. ELM = elementary school version; MS/HS = middle and high school version.

**Table S2.** Completed pre- and post-curriculum surveys by You and Me, Together Vape-Free version and student grade level between January 2025 and June 2025.

|  | Elementary School Version | | Middle/High School Version | |
| --- | --- | --- | --- | --- |
| **Student Grade Level** | Pre  n (%) | Post n (%) | Pre  n (%) | Post n (%) |
| K | 9 (0.2) | 5 (0.1) | 8 (0.0) | 2 (0.0) |
| 1 | 12 (0.3) | 6 (0.2) | 18 (0.0) | 1 (0.0) |
| 2 | 12 (0.3) | 7 (0.2) | 34 (0.1) | 3 (0.0) |
| 3 | 85 (1.9) | 75 (1.9) | 43 (0.1) | 17 (0.1) |
| 4 | 276 (6.2) | 13 (0.3) | 85 (0.2) | 5 (0.0) |
| 5 | 477 (10.7) | 311 (8) | 204 (0.5) | 114 (0.6) |
| 6 | 1,433 (32.1) | 864 (22.2) | 8,615 (22) | 4,353 (22.7) |
| 7 | 2,167 (48.5) | 2,613 (67.1) | 10,164 (25.9) | 4,116 (21.5) |
| 8 | -- | -- | 7002 (17.9) | 3,158 (16.5) |
| 9 | -- | -- | 8642 (22.1) | 5,068 (26.4) |
| 10 | -- | -- | 2,217 (5.7) | 1,145 (6) |
| 11 | -- | -- | 1,043 (2.7) | 534 (2.8) |
| 12 | -- | -- | 1,100 (2.8) | 655 (3.4) |
| College + | -- | -- | 14 (0.0) | 3 (0.0) |
| **Total** | **4,471** | **3,894** | **39,189** | **19,174** |

Note. All pre-post analyses are based on these sample sizes.

**Table S3.** Exploratory linear mixed-effects model results demonstrating changes in pre-post scores moderated by student school grade level. Results are for the middle and high school versions of You and Me, Together Vape-Free.

| **Perceptions of Health Harms** | | | | | | | | | | | | | |
| --- | --- | --- | --- | --- | --- | --- | --- | --- | --- | --- | --- | --- | --- |
|  | **Vape Daily -  Health Harm** | | | | **Vape Daily -  Addiction** | | | **Vape Occasionally - Health Harm** | | | **Vape Occasionally - Addiction** | | |
| Predictor | Est | SE |  | | Est | SE |  | Est | SE |  | Est | SE |  |
| Intercept (B0) | 4.48 | 0.01 | |  | 3.87 | 0.01 |  | 3.78 | 0.01 |  | 3.21 | 0.01 |  |
| Treatment (B1) | 0.09 | 0.01 | |  | 0.19 | 0.02 |  | 0.14 | 0.02 |  | 0.22 | 0.02 |  |
| **Interaction Effects** | |  | |  |  |  |  |  |  |  |  |  |  |
| Grade Level_2 (B0_2) | 0.01 | 0.01 | |  | 0.40 | 0.01 |  | -0.18 | 0.01 |  | 0.23 | 0.01 |  |
| Grade Level_3 (B0_3) | -0.02 | 0.01 | |  | 0.54 | 0.02 |  | -0.32 | 0.01 |  | 0.23 | 0.02 |  |
| Grade Level_4 (B0_4) | -0.18 | 0.02 | |  | 0.42 | 0.03 |  | -0.37 | 0.02 |  | 0.18 | 0.03 |  |
| Treat*Grade Level_2 (B1_2) | -0.02 | 0.02 | |  | -0.11 | 0.03 |  | -0.01 | 0.02 |  | -0.11 | 0.03 |  |
| Treat*Grade Level_3 (B1_3) | 0.05 | 0.02 | |  | -0.08 | 0.03 |  | 0.11 | 0.02 |  | -0.02 | 0.03 |  |
| Treat*Grade Level_4 (B1_4) | 0.13 | 0.03 | |  | -0.01 | 0.05 |  | 0.19 | 0.04 |  | 0.06 | 0.05 |  |
| **Random Effects** |  |  | |  |  |  |  |  |  |  |  |  |  |
| σ² | 0.60 |  | |  | 1.27 |  |  | 0.97 |  |  | 1.23 |  |  |
| 𝛕00 | 0.00 |  |  | | 0.00 |  |  | 0.00 |  |  | 0.00 |  |  |
| **Perceptions of Targeted Marketing** | | | | | | | | | | | | | |
|  | **Youth** | | | | **Black/Brown Communities** | | | **LGBTQ+ Communities** | | | **Adults** | | |
| Predictor | Est | SE |  | | Est | SE |  | Est | SE |  | Est | SE |  |
| Intercept (B0) | 3.45 | 0.01 |  | | 2.93 | 0.01 |  | 2.84 | 0.01 |  | 3.42 | 0.01 |  |
| Treatment (B1) | 0.54 | 0.02 |  | | 0.40 | 0.02 |  | 0.33 | 0.02 |  | -0.06 | 0.02 |  |
| **Interaction Effects** | |  |  | |  |  |  |  |  |  |  |  |  |
| Grade Level_2 (B0_2) | 0.34 | 0.01 |  | | 0.08 | 0.01 |  | 0.01 | 0.01 |  | -0.03 | 0.01 |  |
| Grade Level_3 (B0_3) | 0.45 | 0.01 |  | | 0.16 | 0.01 |  | 0.05 | 0.01 |  | -0.01 | 0.01 |  |
| Grade Level_4 (B0_4) | 0.40 | 0.03 |  | | 0.21 | 0.02 |  | 0.09 | 0.02 |  | 0.05 | 0.02 |  |
| Treat*Grade Level_2 (B1_2) | -0.28 | 0.02 |  | | -0.04 | 0.02 |  | -0.06 | 0.02 |  | 0.06 | 0.02 |  |
| Treat*Grade Level_3 (B1_3) | -0.25 | 0.03 |  | | 0.05 | 0.03 |  | 0.05 | 0.02 |  | 0.15 | 0.02 |  |
| Treat*Grade Level_4 (B1_4) | -0.29 | 0.04 |  | | -0.10 | 0.04 |  | -0.09 | 0.04 |  | 0.09 | 0.04 |  |
| **Random Effects** |  |  |  | |  |  |  |  |  |  |  |  |  |
| σ² | 1.11 |  |  | | 1.03 |  |  | 0.99 |  |  | 0.95 |  |  |
| 𝛕00 | 0.00 |  |  | | 0.00 |  |  | 0.00 |  |  | 0.00 |  |  |
|  | **Additional Perceptions Items** | | | | | | | **Refusal Skills** | | | | | |
|  | **Vapes Safer than Cigarettes** | | | | **Environmental Harm** | | | **E-cigarettes** | | | **Cigarettes** | | |
| Predictor | Est | SE |  | | Est | SE |  | Est | SE |  | Est | SE |  |
| Intercept (B0) | 2.54 | 0.01 |  | | 4.05 | 0.01 |  | 1.57 | 0.01 |  | 1.56 | 0.01 |  |
| Treatment (B1) | -0.28 | 0.02 |  | | 0.25 | 0.02 |  | 0.05 | 0.02 |  | 0.05 | 0.02 |  |
| **Interaction Effects** | |  |  | |  |  |  |  |  |  |  |  |  |
| Grade Level_2 (B0_2) | -0.09 | 0.01 |  | | -0.12 | 0.01 |  | 0.02 | 0.01 |  | 0.00 | 0.01 |  |
| Grade Level_3 (B0_3) | -0.08 | 0.02 |  | | -0.29 | 0.01 |  | 0.07 | 0.01 |  | 0.01 | 0.01 |  |
| Grade Level_4 (B0_4) | -0.01 | 0.03 |  | | -0.38 | 0.02 |  | 0.18 | 0.02 |  | 0.02 | 0.02 |  |
| Treat*Grade Level_2 (B1_2) | 0.12 | 0.02 |  | | -0.03 | 0.02 |  | -0.01 | 0.02 |  | 0.00 | 0.02 |  |
| Treat*Grade Level_3 (B1_3) | -0.03 | 0.03 |  | | 0.09 | 0.02 |  | -0.09 | 0.02 |  | -0.06 | 0.02 |  |
| Treat*Grade Level_4 (B1_4) | -0.09 | 0.04 |  | | 0.19 | 0.04 |  | -0.12 | 0.04 |  | -0.04 | 0.04 |  |
| **Random Effects** |  |  |  | |  |  |  |  |  |  |  |  |  |
| σ² | 1.10 |  |  | | 0.87 |  |  | 0.96 |  |  | 0.91 |  |  |
| 𝛕00 | 0.00 |  |  | | 0.00 |  |  | 0.00 |  |  | 0.00 |  |  |

**Note.** Grade level groups are (1 = 6^th^ grade; 2 = 7-8^th^ grades; 3 = 9-10^th^ grades; 4 = 11-12^th^ grades). Grade level group 1 (6^th^ grade) is the reference group, thus main effects for intercept and treatment slope are specific to this group. Interaction coefficients (e.g., B0_2, B1_2) indicate difference in intercepts and slopes relative to the reference group.

**Table S4.** Logistic regression results indicating different odds of middle/high school post-test completion by student grade level and geographic region.

|  |  |  |
| --- | --- | --- |
| Predictor | OR | CI |
| Intercept | 0.61 | 0.59-0.63 |
| **Grade** |  |  |
| 9th (REF) | -- | -- |
| 5th grade | 0.89 | 0.71-1.13 |
| 6^th^ grade | 0.85 | 0.81-0.90 |
| 7^th^ grade | 0.67 | 0.64-0.70 |
| 8^th^ grade | 0.77 | 0.73-0.81 |
| 10^th^ grade | 0.96 | 0.89-1.04 |
| 11^th^ grade | 0.95 | 0.85-1.06 |
| 12^th^ grade | 1.01 | 0.91-1.12 |
| **Geographic Region** |  |  |
| Pacific-West (REF) | -- | -- |
| Mountain-West | 1.14 | 1.02-1.27 |
| Midwest | 1.05 | 0.99-1.12 |
| South | 0.41 | 0.37-0.45 |
| Northeast | 0.82 | 0.70-0.96 |
| Outside the U.S. | 0.12 | 0.08-0.18 |

Note. OR = odds ratio; CI = 95% confidence interval; REF = reference group. Results based on n = 39,189 pre-tests and n = 19,174 post-tests for the middle/high school version of You and Me.
